# Downstaging of TURBT-Based Muscle-Invasive Bladder Cancer by Radical Cystectomy Predicts Better Survival

**DOI:** 10.5402/2011/458930

**Published:** 2011-04-27

**Authors:** P. R. van Dijk, M. Ploeg, K. K. H. Aben, P. C. Weijerman, H. F. M. Karthaus, J. Th. H. van Berkel, A. C. Viddeleer, A. Geboers, E. van Boven, J. A. Witjes, L. A. L. M. Kiemeney

**Affiliations:** ^1^Department of Urology, Radboud University Nijmegen Medical Centre, 6500 HB Nijmegen, The Netherlands; ^2^Department of Epidemiology, Biostatistics, and HTA, Radboud University Nijmegen Medical Centre, P.O. Box 9101, 6500 HB Nijmegen, The Netherlands; ^3^Department of Cancer Registry and Research, Comprehensive Cancer Centre East, P.O. Box 1281, 6501 BG Nijmegen, The Netherlands; ^4^Department of Urology, Rijnstate Hospital, P.O. Box 9555, 6815 AD Arnhem, The Netherlands; ^5^Department of Urology, Canisius Wilhelmina Hospital, P.O. Box 9015, 6500 GS Nijmegen, The Netherlands; ^6^Department of Urology, St. Jansdal Hospital, P.O. Box 138, 3840 AC Harderwijk, The Netherlands; ^7^Department of Urology, Hospital Gelderse Vallei, P.O. Box 9025, 6710 HN Ede, The Netherlands; ^8^Department of Urology, Slingeland Hospital, P.O. Box 169, 7000 AD Doetinchem, The Netherlands; ^9^Department of Urology, Maas Hospital, P.O. Box 55, 5830 AB Boxmeer, The Netherlands

## Abstract

Differences between clinical (cT) and pathological tumor (pT) stage occur often after radical cystectomy (RC) for muscle-invasive bladder cancer. In order to evaluate the impact of downstaging on recurrence and survival, we selected patients from a large, contemporary, population-based series of 1,409 patients with MIBC. We included all patients who underwent RC (*N=643*) and excluded patients who received (neo)adjuvant therapy, those with known metastasis at time of diagnosis, and those with nonurothelial cell tumors. Disease outcomes were defined as recurrence-free survival (RFS) and relative survival (RS), as a good approximation of bladder cancer-specific survival. After applying the exclusion criteria, 375 patients were eligible for analysis. Tumor downstaging was found to be common after RC; in 99 patients (26.4%), tumor downstaging to non-muscle-invasive stages at RC occurred. Hydronephrosis at baseline and positive lymph nodes at RC occurred significantly less often in these patients. In 62 patients, no tumor was left in the cystectomy specimen. pT stage was pT1 in 20 patients and pTis in 17 patients. Patients with tumor downstaging have about a 30% higher RFS and RS compared to those without. Consequently, tumor downstaging is a favorable marker for prognosis after RC.

## 1. Introduction

The gold standard of treatment for muscle-invasive bladder cancer (MIBC) is radical cystectomy (RC) with pelvic lymph node dissection (PLND). A discrepancy between the initial clinical T-stage (cT-stage) at transurethral resection of the tumor (TURBT) and the final pathological T-stage (pT-stage) after RC is reported in up to 76% of patients [1]. This discrepancy may be due to factors such as poor sensitivity of current imaging, incomplete TURBT with undersampling of muscle tissue, and a long interval between TURBT and RC [2, 3]. Clinical understaging of the tumor is reported in 40–49% of those undergoing RC [1, 4]. Clinical overstaging occurs less often, but still in 22–27% of tumors [1, 5]. Up to 30% of patients with MIBC at TURBT have non-MIBC at RC [5]. These discrepancies between cT-stage and pT-stage have consequences for patient management and counseling.

Several studies have evaluated the effect on prognosis of tumor downstaging from MIBC at TURBT to a nonmuscle-invasive stage at RC. However, these studies report conflicting results: some reported perfect long-term survival rates for patients with tumor downstaging [1, 5–10] while another study reported no survival advantage [11]. Furthermore, the inclusion of patients treated with neoadjuvant systemic chemotherapy and/or radiotherapy [5, 7, 9, 11] and known lymph node metastases at RC [7, 8] complicate the interpretation of the results in these studies.

The current study was performed in order to gain better insight in the impact of downstaging between TURBT and RC on cancer-specific and recurrence-free survival in patients with initial MIBC.

## 2. Materials and Methods

A population-based series of 1,409 patients with MIBC, diagnosed between January 1, 1989 and December 31, 2005, was identified through the population-based cancer registry in the region of the Comprehensive Cancer Centre East in the Netherlands. This cancer centre covers a catchment area of 1.3 million inhabitants and registers all new patients diagnosed with cancer. Bladder cancer is diagnosed and treated in seven centers in this area: one university clinic (Radboud University Nijmegen Medical Centre) and six community hospitals: Canisius Wilhelmina Hospital in Nijmegen, Rijnstate Hospital in Arnhem, Hospital St. Jansdal in Harderwijk, Slingeland Hospital in Doetinchem, Hospital Gelderse Vallei in Ede, and Maas hospital in Boxmeer. After institutional review board approval was obtained, detailed clinical and pathological data of these 1,409 patients were collected retrospectively by reviewing hospital charts between October 2007 and September 2008. Data were collected by use of standardized case record forms. 

For the current study, we included all patients who underwent RC (*N* = 643). To determine the percentage of tumor downstaging, patients who received neoadjuvant therapy (*N* = 111) and patients with unknown pT-stage after RC (*N* = 24) were excluded. 38 patients were excluded because of incomplete data. For further analyses on the characteristics and survival of patients, those with nonurothelial cell tumors (*N* = 80) and patients with known metastasis (N+/M+ disease) at time of diagnosis (*N* = 15) were also excluded. Consequently, 375 patients were available for all analyses.

Maximal cT-stage was determined by pathology examination of the TURBT specimen and imaging (IVU, chest radiography, CT, MRI, and/or bone scans). Pathologic staging was determined by examination of the RC specimen and, when available, lymph nodes derived from the lymph node dissection. 

All specimens were examined by staff pathologists with expertise in genitourinary pathology in four pathology laboratories. The 5th edition (1997) of the International Union Against Cancer (IUCC) [12], TNM staging system was used to classify the cT- and pT stage of all patients. For patients who underwent surgery before 1997, when the 4th edition (1987) was used, we recoded their pathologic stage into the 5th edition. 

Disease outcomes were defined as bladder cancer-specific survival and recurrence-free survival (RFS). Because the cause of death is not available in the cancer registry, the hospital charts and the Municipality Population Registry, bladder cancer-specific survival could not be calculated. Instead, relative survival (RS) was calculated as a good approximation of cancer specific survival [13]. In these analyses the crude survival is adjusted for expected mortality according to annual life tables of the general population according to age, gender, and calendar period distribution. RFS was calculated as the time from RC to the date of the first documented clinical recurrence. If patients had not experienced recurrent disease, they were censored at the date of last followup or time of death. Because we could not retrieve accurate data regarding the RFS for 2 patients, RFS was based on 373 patients.

RS was modeled using a multivariable generalized linear model with an assumed Poisson distribution for the observed number of deaths. RFS was calculated using the Kaplan Meier method. Differences in survival rates were assessed for statistical significance using the log-rank test. 

All analyses of RFS were performed using SPSS version 16.0, Inc, Chicago, Il, USA. The RS was determined using SAS version 9.1 (Cary, NC).

## 3. Results

In total, 643 patients with MIBC received RC. After exclusion of the neoadjuvant treated patients, and the patients with incomplete data, 470 patients remained. In 22.8% (*N* = 107) of these patients the tumor showed downstaging to a nonmuscle invasive stage. As described before, for subsequent analyses patients with non-UC and metastasized disease were also excluded.

### 3.1. Clinical and Pathological Characteristics of the Total Study Population

The study population consisted of 375 patients. Baseline characteristics are presented in Table 1. Median age of the total study population was 68.3 years. The male:female ratio was 3.3 : 1. For 344 patients, the maximal cT-stage was based on TURBT. 31 patients had a higher cT-stage based on imaging techniques.

Pathological evaluation of the cystectomy specimen showed muscle-invasion in 276 (73.6%) patients while 99 (26.4%) patients were downstaged to a nonmuscle invasive stage (i.e., pT0, pTis, or pT1). The presence of hydronephrosis was the only precystectomy variable that showed significant difference between both groups. In the downstaged group 12.1% of patients had hydronephrosis compared to 28.3% in the group of patients without tumor downstaging (*P* = .032). 

In the group of patients with tumor downstaging, 62 (16.5%) patients had no tumor left in their RC specimen (pT0), 20 (5.3%) had pT1, and 17 (4.5%) had pTis. 4 (4.0%) patients (2 pTis and 2 pT1) had positive lymph nodes (pN+) at RC, 60 (60.6%) had negative lymph nodes (pN0) and for 35 (35.4%) patients, the N-status was unknown. Among cases without tumor downstaging these numbers were 46 (16.7%), 155 (56.2%), and 75 (27.1%) patients, respectively. 

### 3.2. Survival Analysis

**Figure 1:**
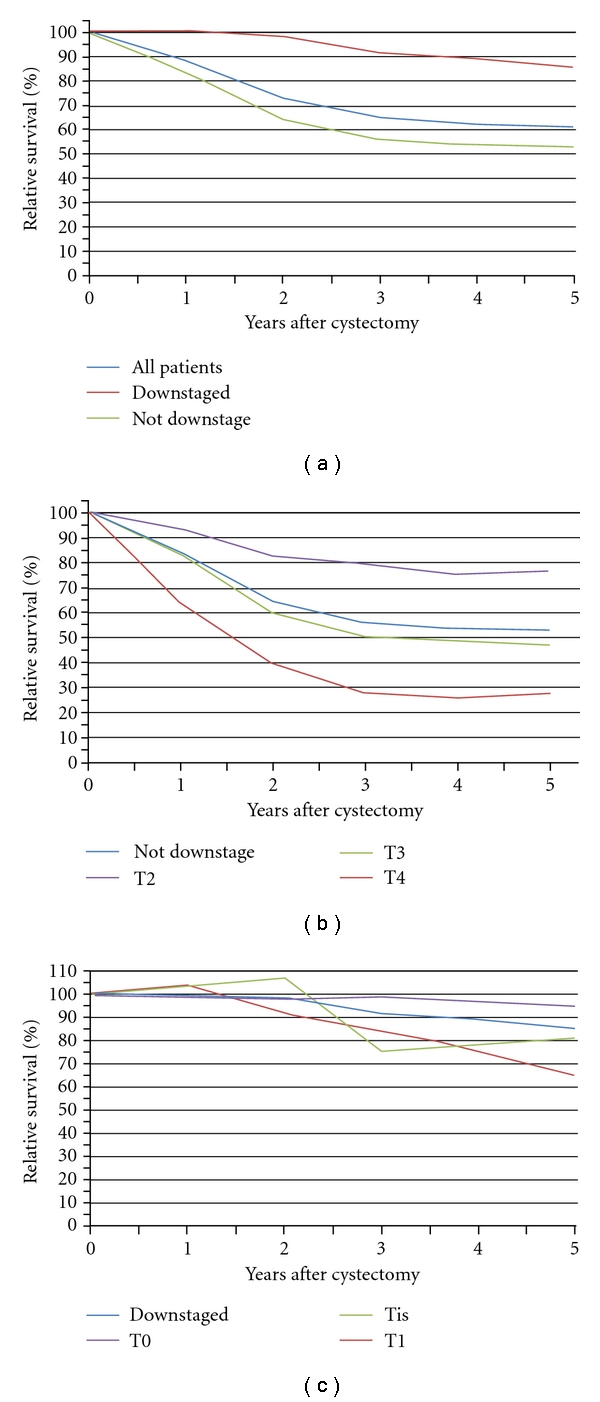
Kaplan-Meier curves of the relative survival (RS) for: (a) all patients, downstaged and not downstaged patients, (b) Not downstaged patients and patients with a pT2, pT3, and pT4 stage, and (c) Downstaged patients and patients with a pT0, pTis, and pT1 stage.

Median followup time for all patients was 7.7 years (range 0.9–19.0).  Table 2 and Figure 1 show the 1-, 3-, and 5-year RS rates for all studied patients. The 5-year RS for patients with a downstaged tumor was 84.9% compared to 53.4% for patients who appeared to have muscle-invasion in the RC specimen (*P* < .001). Among the patients with tumor downstaging, those who had no tumor left showed the best RS at 5 years after surgery: 93.9% compared to 64.7% and 80.7% for patients with a pT1 stage (*P* = .042) and pTis stage (*P* = .25), respectively. 

Disease recurrence occurred in 137 (36.5%) of 375 patients. Of these patients 12 had a downstaged tumor and 125 had a muscle-invasive tumor at RC. As depicted in Table 2, patients in whom disease was downstaged from ≥cT2 to a nonmuscle-invasive tumor stage at RC demonstrated a RFS of 85.2% compared to 49.9% for patients who had a muscle-invasive tumor at RC (*P* < .001). The 12 patients who suffered from recurrences were equally distributed over the different stages (pT0, pT1, pTis). The 5-year RFS of the total population with downstaged tumors (*N* = 99) was 85.2%. After 5 years of followup 91.9%, 78.6%, and 69.6% of these patients with a pT0, pT1, and pTis stage were free of recurrence. Although the best RFS was demonstrated by patients with a pT0 tumor stage, the differences among downstaged patients did not reach statistical significance.

## 4. Discussion

The current study provides insight in the survival of patients with MIBC downstaged to a nonmuscle-invasive tumor stage or pT0 after TURBT. All patients were treated by RC and were unexposed to any kind of (neo)adjuvant therapy. In 26.4% of these patients the tumor was downstaged. Not surprisingly, patients who experienced tumor downstaging showed a significantly better survival compared to patients with residual muscle-invasive tumor stages at RC. Patients who did not demonstrate downstaging were at highest risk of recurrence and mortality. They showed a RFS of 49.9% and a RS of 53.4% at 5 years. Among patients with tumor downstaging those who had no tumor left showed the best RS and RFS of 93.9% and 91.9%, respectively, at 5 years after surgery. For patients with tumor downstaging to pT1, and pTis, the RS at 5 years was 64.7% and 80.7%, respectively and the RFS at the same time point was 78.6% and 69.6%, respectively. 

A discrepancy between clinical and pathologic staging is a common finding in clinical practice with prognostic consequences that have been described in previous reports. Nielsen et al. [7] assessed RFS among patients noted to have MIBC on TURBT but were subsequently found to have NMIBC at RC. Of the 112 patients, 25 (22.3%) were downstaged to non-MIBC at RC. They demonstrated a significantly better RFS for downstaged patients (84.0%) compared to those who were not downstaged (66.7%). Even after adjusting for lymph node status or chemotherapy in a multivariable model, a threefold reduction in recurrence risk with downstaging was seen. Shariat et al. [5] studied a group of 490 patients who had a muscle-invasive cT-stage and were treated with or without (neo)adjuvant therapy and RC. They found that patients who had nonmuscle invasive pathological stage showed a lower probability of bladder cancer recurrence and disease-specific mortality than patients who remained muscle-invasive [5]. Recently, Isbarn et al. [6] described that in 208 patients with cT2N0 stage bladder cancer a lower T-stage at RC correlated with improved outcomes relative to those with pT2N0 disease. Patients who received peri-operative chemotherapy were excluded from their analysis. When all downstaged cases were grouped (pT0, pTa, and pT1) a significantly better recurrence-free and bladder cancer specific survival was found for these patients compared to pT2 cases. In addition, they proposed a trend effect on survival according to the extent of TURBT downstaging. Except for a better RS for patients with a pT0 stage after RC compared to a pT1 stage there were no significant differences in survival among the downstaged patients in our study. Probably these groups of patients were too small (*N* = 62, *N* = 20, and *N* = 17 for pT0, pT1, and pTis, resp.) to detect a significant intragroup difference. However, both the RS and the RFS among patients with tumor downstaging showed a trend towards a better survival according to the extent of the invasiveness and volume of the tumor. Data from Herr [14] also suggest that downstaging by TURBT can offer a survival benefit. They presented the outcomes of patients with MIBC treated by TURBT only. The disease specific survival rate in their series for patients with stage cT0 on restaging TURBT was 82% compared to 57% for patients with cT1 disease on restaging TURBT (*P* = .003). In opposition, in a more historical series, Thrasher et al. found no improved outcome after tumor downstaging. They examined the survival advantage of a stage pT0 in 66 of 433 patients (213 had clinical MIBC) who underwent RC for bladder cancer. They found that patients with a pT0 cystectomy specimen did not have a survival advantage over those who had similar cT- and pT-stage [11]. 

Although patients with tumor downstaging to a pT0 stage had the best RS and RFS there was still disease attributable mortality in this group of patients. Apart from the possibility that the pathologic pT0 stage was inaccurate or the recurrences were of other origin than the bladder, the most logical explanation for this observation is the occurrence of undetected metastases [10]. In the population evaluated by Isbarn et al. there were no recurrences or bladder cancer specific deaths after 5 years of followup among the patients downstaged to pT0 stage, which is in conflict with the RS and RFS of pT0N0 patients found in our study (93.9% and 91.9%, resp.). Taken together, these findings suggest that, although patients with a pT0 tumor constitutes a subgroup with the best survival among patients with tumor downstaging, having no tumor left in the cystectomy specimen may still come along with a small excess of bladder cancer deaths.

The use of neoadjuvant chemotherapy for cT2 disease is still under debate. In its most recent guideline the European Association of Urology indicates that neoadjuvant cisplatin-based combination chemotherapy should be considered in MIBC [15]. As mentioned before, Isbarn et al. found a 5-year RFS and cancer specific mortality of both 100% for patients downstaged to pT0, pTa and pTis. The authors concluded that neoadjuvant chemotherapy may not be necessary nor beneficial in cT2 patients downstaged to pT0, pTa or pTis by TURBT. However, the RS and RFS for pT0 and pTis after 5 years of followup (93.9% and 91.9% for pT0, 80.7 and 69.6% for pTis) in our study does not underline this statement. Apart from the fact that it is not possible to predict the degree of tumor downstaging after TURBT, the recommendation to withhold neoadjuvant chemotherapy for all cT2 patients after a complete TURBT cannot be taken based on the completeness of a TURBT alone.

Our finding that radical resection of MIBC at TURBT resulting in a nonmuscle invasive tumor stage or pT0 at RC is associated with improved survival may have several explanations. Complete resection at TURBT may independently provide a protective effect on outcome. On the other hand it is possible that initially less deeply invasive or smaller tumors before TURBT and, thus, lower residual tumor volume after TURBT can confound the benefit of TURBT related downstaging [6]. We found that patients with tumor downstaging showed less often hydronephrosis. This may suggest that patients with tumor downstaging had less often deeply invasive tumors and/or a lower tumor volume at time of diagnosis. 

Our findings suggest that a thorough and complete TURBT may be warranted in most patients who are candidates for RC and have MIBC at TURBT. However, such recommendations may not apply to patients with tumors >T2 tumors in whom complete TURBT may lead to perforation and its consequences.

This study has some limitations. First, cause of death was not available. Therefore, RS analyses were used to approximate the cancer-specific survival. Because relative survival analysis does not adjust for smoking habits, the use of RS may have slightly overestimated bladder cancer mortality. Furthermore, our study has limitations inherent to retrospective analysis: the TURBT and cystectomy procedures were performed by different surgeons, the specimens were evaluated by multiple pathologists overtime, and central review of pathology was not possible. This might have caused some misclassification of staging and additional variability in prognosis after cystectomy. On the other hand, it reflects real-life urologic practice.

## 5. Conclusions

Tumor downstaging is a common finding; 26.4% of patients with a clinical muscle-invasive tumor were downstaged to a nonmuscle-invasive stage. Patients with tumor downstaging have a ~30% better recurrence-free survival and relative survival compared to those without. Consequently tumor downstaging is probably the most favorable marker for prognosis after RC.

## Figures and Tables

**Table 1:** Baseline characteristics of the total study population.

Variable	Total (*N* = 375)	Downstaged (*N* = 99)	Not downstaged (*N* = 276)
	Precystectom *N*	*N*	*N*
Population sex (%)			
Total	375	99	276
Male	287 (76.5)	77 (78.8)	210 (76.1)
Female	88 (23.5)	22 (22.2)	66 (23.9)
History of other malignancy (%)	63 (16.8)	21 (21.2)	42 (16.3)
Comorbidity (%)	235 (62.3)	58 (58.6)	177 (64.1)
Hydronephrosis (%)	90 (24.0)	12 (12.1)*	78 (28.3)*
Tumor grade TURBT specimen (%)			
I	0 (0.3)	0 (0.0)	1 (0.4)
II	41 (10.9)	15 (15.2)	26 (9.4)
III	304 (81.1)	74 (74.7)	230 (83.3)
Unknown	29 (7.7)	10 (10.1)	19 (6.9)
Concomitant CIS in TURBT specimen (%)	74 (19.7)	25 (25.2)	49 (17.8)

Median age at cystectomy in years (range)	68.3 (33.6–94.6)	66.4 (36.0–84.2)	68.6 (33.6–94.6)
Median time between TURBT and RC in days (range)	43.8 (3.7–412.5)	47.5 (3.7–127.8)	43.8 (3.7–412.5)
T-stage RC specimen			
T0	62 (16.5)		
T1	20 (5.3)		
Tis	17 (4.5)		
≥T2	276 (73.6)		
Grade (%)			
I	1 (0.3)	0 (0.0)	1 (0.4)
II	24 (6.4)	2 (2.0)	22 (8.0)
III	234 (62.4)	19 (19.2)	215 (77.9)
None	62 (16.5)	62 (62.6)	0 (0)
Unknown	54 (14.4)	16 (16.2)	38 (13.7)
N-stage PLND specimen (%)			
N0	215 (57.3)	60 (60.6)	155 (56.2)
N+	50 (13.3)	4 (4.0)*	46 (16.7)*
Nx	110 (29.4)	35 (35.4)	75 (27.1)
Concomitant CIS in RC specimen (%)	106 (28.3)	26 (26.3)	80 (29.0)

*Chi-square test: *P* < .05.

**Table 2:** Relative survival (RS) and recurrence free survival (RFS) after 1, 3, and 5 years of followup.

Survival	Group	*N*	1 year (±SE)	3 years (±SE)	5 years (±SE)
RS	All	375	88.4 (1.9)	65.8 (2.8)	61.7 (3.1)

	Downstaged	99	100.7 (1.5)	91.3 (4.2)	84.9 (5.6)
	T0	62	99.0 (2.3)	98.3 (3.9)	93.9 (6.2)
	T1	20	103.8	84.1 (10.9)	64.7 (13.5)
	Tis	17	103.1	75.0 (13.1)	80.7 (14.1)
	Not Downstaged	276	84.0 (2.4)	56.7 (3.3)	53.4 (3.6)
	T2	84	93.0 (3.3)	79.3 (5.3)	76.0 (6.1)
	T3	160	83.0 (3.2)	50.4 (4.4)	46.6 (4.7)
	T4	32	65.3 (8.9)	28.2 (8.8)	27.5 (9.6)

RFS	All	375	77.2 (2.2)	61.3 (2.7)	59.6 (2.7)

	Downstaged	99	97.9 (1.5)	88.7 (3.4)	85.2 (4.0)
	T0	62	100	94.3 (3.2)	91.9 (3.9)
	T1	20	89.5 (7.0)	78.6 (9.5)	78.6 (9.5)
	Tis	17	100	81.2 (9.8)	69.6 (13.6)
	Not Downstaged	276	69.4 (2.9)	51.0 (3.2)	49.0 (3.2)
	T2	84	84.8 (4.0)	72.7 (5.1)	70.7 (5.3)
	T3	160	64.0 (3.9)	42.3 (4.2)	42.3 (4.2)
	T4	32	51.2 (10.0)	26.5 (9.7)	26.1 (9.7)

## References

[1] Hollenbeck BK, Miller DC, Dunn RL, Montie JE, Wei JT (2005). The effects of stage divergence on survival after radical cystectomy for urothelial cancer. *Urologic Oncology: Seminars and Original Investigations*.

[2] Herr HW (1999). The value of a second transurethral resection in evaluating patients with bladder tumors. *Journal of Urology*.

[3] Chang SS, Hassan JM, Cookson MS, Wells N, Smith JA (2003). Delaying radical cystectomy for muscle invasive bladder cancer results in worse pathological stage. *Journal of Urology*.

[4] Pagano F, Bassi P, Galetti TP (1991). Results of contemporary radical cystectomy for invasive bladder cancer: a clinicopathological study with an emphasis on the inadequacy of the tumor, nodes and metastases classification. *Journal of Urology*.

[5] Shariat SF, Palapattu GS, Karakiewicz PI (2007). Discrepancy between clinical and pathologic stage: impact on prognosis after radical cystectomy. *European Urology*.

[6] Isbarn H, Karakiewicz PI, Shariat SF (2009). Residual pathological stage at radical cystectomy significantly impacts outcomes for initial T2N0 bladder cancer. *Journal of Urology*.

[7] Nielsen ME, Bastian PJ, Palapattu GS (2007). Recurrence-free survival after radical cystectomy of patients downstaged by transurethral resection. *Urology*.

[8] Volkmer BG, Kuefer R, Bartsch G (2005). Effect of a pT0 cystectomy specimen without neoadjuvant therapy on survival. *Cancer*.

[9] Palapattu GS, Shariat SF, Karakiewicz PI (2006). Cancer specific outcomes in patients with pT0 disease following radical cystectomy. *Journal of Urology*.

[10] Loizaga Iriarte A, Senarriaga Ruiz de la Illa N, Lacasa Viscasillas I, Rábade Ferreiro A, Iriarte Soldevilla I, Unda Urzaiz M (2009). Does a pT0 cystectomy specimen imply being tumour-free in the long term?. *Actas Urologicas Espanolas*.

[11] Thrasher JB, Frazier HA, Robertson JE, Paulson DF (1994). Does a stage pT0 cystectomy specimen confer a survival advantage in patients with minimally invasive bladder cancer?. *Journal of Urology*.

[12] International Union against Cancer (IUCC) (2009). *TNM Classification of Malignant Tumors*.

[13] Dickman PW, Sloggett A, Hills M, Hakulinen T (2004). Regression models for relative survival. *Statistics in Medicine*.

[14] Herr HW (2001). Transurethral resection of muscle-invasive bladder cancer: 10-year outcome. *Journal of Clinical Oncology*.

[15] Stenzl A, Cowan NC, De Santis M Guidelines on bladder cancer: muscle invasive and metastatic.

